# Fuzzy Union to Assess Climate Suitability of Annual Ryegrass (*Lolium multiflorum*), Alfalfa (*Medicago sativa*) and Sorghum (*Sorghum bicolor*)

**DOI:** 10.1038/s41598-018-28291-3

**Published:** 2018-07-05

**Authors:** Hyunae Kim, Shin Woo Hyun, Gerrit Hoogenboom, Cheryl H. Porter, Kwang Soo Kim

**Affiliations:** 1 Institute of Convergence Technology, KT, Seoul, 06763 Korea; 2 Department of Plant Science, Seoul National University, Seoul, 08826 Korea; 3 Department of Agricultural and Biological Engineering, University of Florida, Gainesville, Florida, 32611 USA; 4 Institute for Sustainable Food Systems, University of Florida, Gainesville, Florida, 32611 USA; 5 Research Institute of Agriculture and Life Sciences, Seoul National University, Seoul, 08826 Korea

## Abstract

The Law of the Minimum is often implemented using t-norm or fuzzy intersection. We propose the use of t-conorm or fuzzy union for climate suitability assessment of a grass species using annual ryegrass (*Lolium multiflorum* Lam.) as an example and evaluate the performance for alfalfa (*Medicago sativa* L.) and sorghum (*Sorghum bicolor* L.). The OR_F_ and AND_F_ models, which are fuzzy logic systems based on t-conorm and t-norm between temperature and moisture conditions, respectively, were developed to assess the quality of climate conditions for crops. The parameter values for both models were obtained from existing knowledge, e.g., the EcoCrop database. These models were then compared with the EcoCrop model, which is based on the t-norm. The OR_F_ model explained greater variation (54%) in the yield of annual ryegrass at 84 site-years than the AND_F_ model (43%) and the EcoCrop model (5%). The climate suitability index of the OR_F_ model had the greatest likelihood of occurrence of annual ryegrass compared to the other models. The OR_F_ model also had similar results for alfalfa and sorghum. We emphasize that the fuzzy logic system for climate suitability assessment can be developed using knowledge rather than presence-only data, which can facilitate more complex approaches such as the incorporation of biotic interaction into species distribution modeling.

## Introduction

Assessment of climatic suitability of a crop could help introduce a new crop into a cropping system as an adaptation option to climate change^1–4^. A process-based model that quantifies photosynthetic assimilates under given weather conditions could be used to examine climatic suitability of a crop^5–7^. Still, quantitative prediction of crop growth would require a detailed set of input data for a process-based model. For example, input regarding local management information such as variety, fertilizer application dates and irrigation application rates, is required for the process-based model although such information is often limited to specific sites^8^. Furthermore, high quality observed data would be needed for calibration and evaluation of the model^9–11^.

When data availability is limited to occurrence sites of a given species, a species distribution model would be useful for the assessment of climate suitability in a region^12–14^. For example, Estes *et al*.^15^ reported that an empirical model based on the maximum entropy had similar or better accuracy compared with a process-based model in predicting productivity of maize. Jeong *et al*.^16^ used the random forest algorithm to predict wheat yield at different spatial scales. However, these approaches require a number of sites to find the relationship between bioclimatic variables and climate suitability of a given species.

A fuzzy logic approach could allow for the development of a climate suitability model for a species using human expert knowledge rather than occurrence data. Models based on fuzzy logic have been developed to predict the distribution of forest herbs^17^, to identify optimum management of rangeland^18^, and to analyze cropland suitability^19^. as well as environmental studies^20–22^. Fuzzy logic models have been developed using an optimization process, e.g., a genetic algorithm, that depends on training data, which would result in a black box model^23–25^. In contrast, the fuzzy logic model that has the high-level of interpretability can be developed using knowledge based on the relationship between environmental conditions and growth of the species of interest^26^.

The Law of the Minimum could provide a reasonable framework to assess climate suitability of crops using a fuzzy logic model^27^. Fuzzy propositions such as “temperature is suitable” and “precipitation is suitable” can be defined to interpret the Law of the Minimum for climate variables. Literally, the Law of the Minimum is the outcome of fuzzy intersection using the minimum t-norm between those propositions. For example, Hackett & Vanclay^28^ used the minimum value of indices to estimate growth of plant using the law of the Minimum. However, the Law of the Minimum can be represented using fuzzy intersection based on other t-norms such as the product t-norm to represent the minimum quality of given conditions. For example, Hijmans *et al*.^29^ developed the EcoCrop model that assesses climate suitability of crops using the product between suitability indices of temperature and precipitation. The EcoCrop model has been used for climate change impact assessment for minor crops^30^.

Austin^31^ suggested that the Law of the Minimum could have limitations in development of climate suitability models. In particular, a climate suitability model can be developed using climate variables that account for a small variation in plant growth. For example, the EcoCrop model is dependent on annual precipitation (Appendix A in Supporting information) although growth of a grass could be affected by the rainfall distribution during a growing season^32^. The species parameter values for the EcoCrop model can be obtained from the FAO EcoCrop database, which would require no iterative calibration process. Still, Ramirez-Villegas *et al*.^33^ found that the EcoCrop model did not have a clear relationship between the climate suitability index and sorghum yield even after parameter optimization for the crop. Piikki *et al*.^34^ improved the EcoCrop model using local soil properties after additional optimization procedures.

Here we propose an algorithm based on a fuzzy logic system to assess climate suitability of a forage crop. We attempted to examine if climate suitability assessment could be improved using an alternative rule based on fuzzy union or t-conorm to the Law of the Minimum instead of an iterative calibration process. The objectives of this study were to develop and evaluate the fuzzy systems based on fuzzy intersection and fuzzy union. We compared the fuzzy logic system based on t-conorm (fuzzy union) with the other model based on t-norm (fuzzy intersection) for logical operation between suitability indices of temperature and rainfall. Then, we compared these models with the EcoCrop model, which is based on the fuzzy intersection. The parameter values for the fuzzy logic model were determined using the EcoCrop database to have identical parameter values for both models. In the present study, annual ryegrass (*Lolium multiflorum* Lam.) was selected because it has been considered an important forage crop in temperate regions^35,36^ yet limited modeling efforts so far have been conducted. Furthermore, the feasibility of the proposed algorithm for the climate suitability indices was evaluated for alfalfa (*Medicago sativa* L.), a cool season legume crop, and sorghum (*Sorghum bicolor* L.), a warm season grass, respectively.

## Materials and Methods

### Rules to evaluate climate conditions for survival and growth of a plant species

Climate suitability of a species has been modelled using a combination of rules that characterizes the environmental envelopes of the given species. Models often depend on the t-norm, which generalizes intersection in a logic operation between two environmental conditions such as temperature and precipitation^33,37,38^. The EcoCrop model evaluates rules that could be described as “temperature is suitable *and* precipitation is suitable.” The use of a t-norm would take into account the minimum quality of environmental conditions as the Law of the Minimum indicates^27,39^. The t-norm would be useful to determine the boundary conditions favorable for the survival of a given species^40^.

Alternatively, climate conditions for growth as well as survival of a species could be assessed using a t-conorm, which is a logical union operation^37^. When the quality of environmental conditions is greater than the threshold of survival for a species, the species would have additional growth performance at the degree to which these conditions meet the requirements for optimum growth of the species. For example, a long duration of optimum temperature would have a positive impact on plant growth unless drought conditions are prolonged. Although a temperature condition during a month could be suboptimal, a grass species could benefit from the optimum amount of rainfall in a given month. Thus, a rule based on t-conorm, e.g., “temperature is suitable *or* precipitation is suitable,” could be useful for delineating factors in the assessment of climate suitability of the given species.

Rule statements were designed to represent survival and growth of a grass species in terms of precipitation and temperature (Table 1). Growth of the species was assessed using a statement representing the moisture conditions under favorable temperature conditions. For example, a statement such as “rainfall amount is adequate for the time period during which temperature conditions would be favorable,” would be useful to represent a moisture condition for growth of a species. The rule statement was rephrased as “rainfall is suitable, maximum temperature is favorable, and minimum temperature is favorable.”

**Table 1:** Rule statements to evaluate the climate suitability for a grass species.

Rule	Statement	Eq.*	Variable
1	Precipitation is suitable and Maximum Temperature is favorable and Minimum Temperature is favorable	15	β
2	Temperature is suitable for a less dry period	16	Θ
3	Maximum Temperature is stressful or Minimum Temperature is stressful	19	*τ*
4	Maximum Temperature is harmful or Minimum Temperature is harmful	20	ξ

*Eq. is the equation number in the text.

Another rule was defined to represent the temperature suitability for growth of a species (Table 1). At first, the rule statement, “temperature is suitable,” was used to determine the degree to which temperature conditions were suitable for growth of a species. Then, a modifier, “for a less dry period” was added to the predicate “suitable” to take into account a negative impact of dry conditions for consecutive months on crop growth.

Additional rule statements were defined to take into account suboptimal and extreme conditions (Table 1). The rules of heat and cold stresses including “maximum temperature is stressful” and “minimum temperature is stressful,” respectively, were used to represent the negative impact of suboptimum temperature conditions on plant growth. Extreme temperature conditions during a season, which would affect survival of a species, were also characterized using rule statements such as “maximum temperature was harmful” and “minimum temperature was harmful.” In addition, the rule statement “temperature was reasonable for early growth” was used to evaluate whether or not a given species could be established at the beginning of a season.

### Evaluation of rule statements

**Figure 1:**
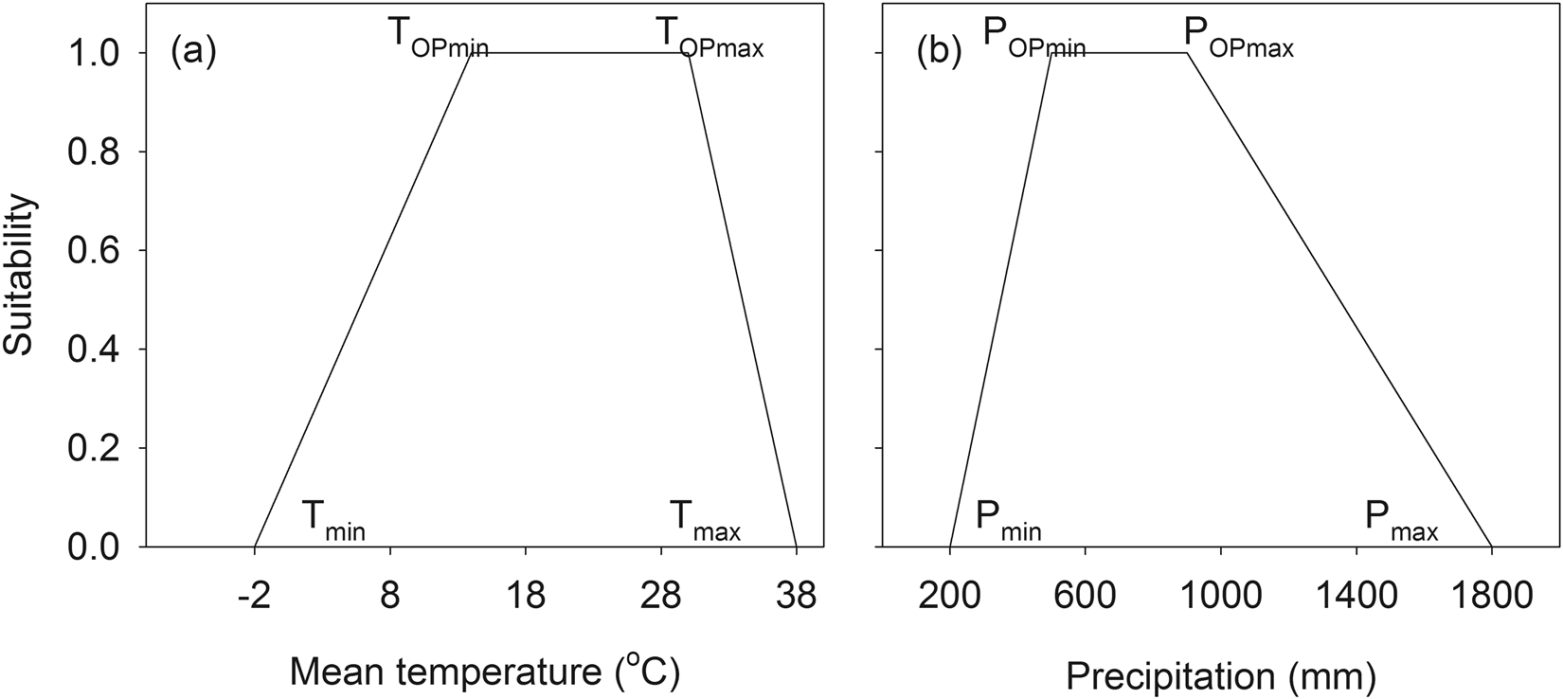
Examples of membership functions for temperature (**a**) and precipitation (**b**). These same functions were used to determine the suitability of temperature and precipitation for the EcoCrop model.

Each rule statement was evaluated using a linguistic term of the predicates, which represents specific conditions for survival or growth of a species. Using a fuzzy set, each linguistic term was quantified for a corresponding climate variable using a crisp value of the given variable. A fuzzy set is a set whose members have a degree of membership between 0 and 1 instead of bivalent membership such as yes or no^41^. A membership function assigns a degree of membership to the value of a given variable. For example, the proposition “temperature is suitable” can be evaluated to have the degree of truth, e.g., 0.75, for a mean temperature in a month, e.g., 11 °C, at a site using a membership function illustrated in Fig. 1a. Similarly, suitability of precipitation is also determined using another membership function (Fig. 1b). A detailed description of fuzzy sets can be found in Klir & Yuan^37^.

**Figure 2:**
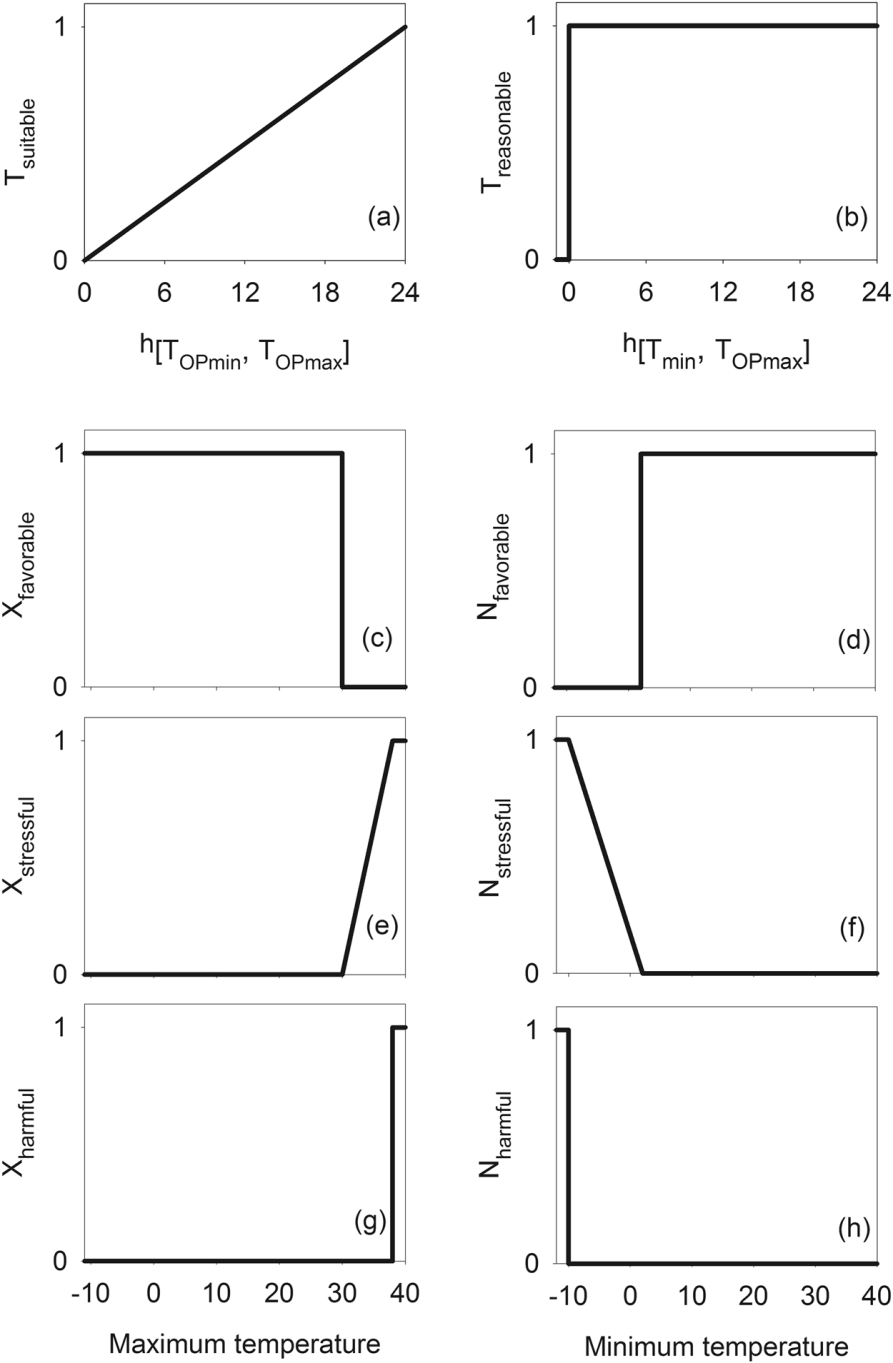
Membership functions of the model based on a fuzzy logic system. *X* and *N* represent the membership function that uses maximum and minimum temperature as inputs, respectively. *h*_*(a,b)*_ indicates the number of hours during which the temperature ranged between a and b.

Fuzzy sets for each linguistic term were defined using different shapes (Fig. 2; Appendix B). The shape of fuzzy sets was designed to minimize the number of parameters and to maximize the existing knowledge on climate envelope. For example, a trapezoid shape was used to evaluate suitability of precipitation because the ranges of precipitation can be obtained from the EcoCrop database (Fig. 1b). The degree of temperature suitability was determined using a right triangle shape based on the duration of hours during which the optimal temperature conditions were met (Fig. 2a).

The combination of predicates defined in a rule statement was evaluated using fuzzy operations. The connectives in the rule statements, *and* and *or*, were defined using a t-norm and a t-conorm, respectively, as follows:

1 $${F}_{x}\,and\,{F}_{y}={F}_{x}\cdot {F}_{y}\,and$$

2 $${F}_{x}\,or\,{F}_{y}={F}_{x}+{F}_{y}-{F}_{x}\cdot {F}_{y},$$

where *F* ∈ [0, 1]. The rule statements for moisture and temperature suitability were rewritten as follows:

3 $$\beta ({X}_{m},\,{N}_{m},{P}_{m})={P}_{suitable}({P}_{m})\cdot {X}_{favorable}({X}_{m})\cdot {N}_{favorable}({N}_{m}),$$

4 $${\rm{\Theta }}({X}_{m},\,{N}_{m},\,{d}_{m})={{d}_{m}}^{-1}\cdot {T}_{suitable}({X}_{m},\,{N}_{m}),$$

where β, *Θ*, and *d* represent the outcome of rule evaluation for moisture, temperature suitability, and a weight for a prolonged dry condition respectively. The value of *d* was determined to be the number of consecutive months during which β value was zero by the time of evaluation. *P*_*suitable*_, *X*_*favorable*_, *N*_*favorable*_, and *T*_*suitable*_ are the membership function of fuzzy sets for suitability of precipitation, favorability of maximum temperature, favorability of minimum temperature, and suitability of temperature in a month, respectively (Fig. 2). *P*_*m*_, *X*_*m*_, and *N*_*m*_ indicate precipitation, maximum temperature, and minimum temperature in a month *m*, respectively.

### Calculation of climate suitability index

The architecture of the models based on t-norm and t-conorm was designed to evaluate cumulative climate quality during plant growth. For example, the outcome of rules evaluated by each month was accumulated over a growing period of a species. The architecture for the fuzzy models differed by that of the EcoCrop model because the latter determines climate suitability by selecting the minimum climate quality during the growing period. As a result, the architecture used by the fuzzy models would allow for a reasonable evaluation of the conditions for plant growth compared with the EcoCrop model although both models depend on monthly climate conditions. However, it is likely that the fuzzy models would have inferior prediction of plant growth compared to the process-based crop models that rely on more detailed input data and parameters^8–11^. Nevertheless, the fuzzy model could be useful to assess climate conditions for crop growth when detailed information on crop management and environmental conditions are rarely available or a detailed crop model does not exist, such as for many vegetable, fruit and ornamental crops.

The outcomes of rule evaluation for moisture and temperature suitability were combined using two types of logical operations, t-norm or t-conorm. To represent the Law of the Minimum, the rule statements were combined using a t-norm, *S*_*AND*_, as follows:

5 $${S}_{AND}({X}_{m},\,{N}_{m},\,{P}_{m},\,{d}_{m})={\rm{\Theta }}({X}_{m},\,{N}_{m},\,{d}_{m})\cdot {\rm{\beta }}({X}_{m},\,{N}_{m},\,{P}_{m})$$

We used the standard intersection t-norm because it provides a low value for suitability indices, which would be useful to represent the Law of the Minimum in practice. Climate suitability assessed using the standard union t-conorm, *S*_*OR*_, was also defined as follows:

6 $${S}_{OR}({X}_{m},\,{N}_{m},\,{P}_{m},\,{d}_{m})={\rm{\Theta }}({X}_{m},\,{N}_{m},\,{d}_{m})+{\rm{\beta }}({X}_{m},\,{N}_{m},\,{P}_{m})-{\rm{\Theta }}({X}_{m},\,{N}_{m},\,d)\cdot {\rm{\beta }}({X}_{m},\,{N}_{m},\,{P}_{m})$$

Models based on t-conorm and t-norm were denoted by OR_F_ and AND_F_ models, respectively.

Climate suitability index in a season was determined by combining the degrees of cumulative suitability and stresses. The monthly stress condition *τ* was determined as follows:

7 $$\tau ({X}_{m},\,{N}_{m})={N}_{stressful}({N}_{m})+{X}_{stressful}({X}_{m})-{N}_{stressful}({N}_{m})\cdot {X}_{stressful}({X}_{m}),$$

where *X*_*stressful*_ and *N*_*stressful*_ indicate the membership function of “stressful” for maximum and minimum temperatures, respectively (Fig. 2e,f). It was assumed that the stress would have a long-term memory on the climate suitability in following months depending on the magnitude of stress conditions. Thus, the degree of cumulative monthly suitability *Φ*_*m*_ was calculated as follows:

8 $${{\rm{\Phi }}}_{m}=\,{\rm{\max }}({{\rm{\Phi }}}_{m-1}+{S}_{m}-{\tau }_{m},\,0),$$

where *S*_*m*_ and *τ*_*m*_ denote *S(X*_*m*_*, N*_*m*_*, P*_*m*_*, d*_*m*_) and *τ(X*_*m*_*, N*_*m*_). If the value of *Φ*_*m*_ was less than 0, it was set to be zero under the assumption that growth would be ceased by climatic stresses. The value of *Φ*_0_ was assumed to be zero.

The climate suitability index was calculated for a growing period. For the growing period, climate suitability would be dependent on occurrence of extreme conditions and favorable conditions for emergence. The suitability index *G*_*p*_ for a growing period *p* was calculated as follows:

9 $${G}_{p}={T}_{reasonable}({X}_{0},\,{N}_{0}){.{\rm{\Phi }}}_{p}/{n}_{p}\cdot \prod _{m}(1-\kappa ({X}_{m},{N}_{m})),$$

where *Φ*_*p*_ and *n*_*p*_ indicate the suitability index and the number of month in *p*, respectively. The extreme conditions *κ* against survival of a given species in *m* was determined as follows:

10 $$\kappa ({X}_{m},\,{N}_{m})={X}_{harmful}({X}_{m})+{N}_{harmful}({N}_{m})-{X}_{harmful}({X}_{m})\cdot {N}_{harmful}({N}_{m}),$$

where *X*_*harmful*_ and *N*_*harmful*_ represent the membership function of “harmful” for maximum and minimum temperatures, respectively (Fig. 2f,g). The product of degree to which monthly conditions were not harmful, i.e., 1-*κ(X*_*m*_*, N*_*m*_), was used to take into account only the growing period during which no extreme condition occurred. *T*_*reasonable*_ represents the membership function of “reasonable” for establishment in an early season (Fig. 2b). *T*_*reasonable*_ was also used to avoid calculating the climate suitability index at sites where climate condition would be unfavorable for establishment of a given species during the first month.

The length of a growing period can vary by region and management. When no harvest date was available, multiple values of climate suitability index were calculated for a set of potential growing periods within a growing season. When a growing season started in March and the length of a growing period for a crop would range from 3–5 months, the values of *G*_*p*_ were determined for a period from March to May, March to June, and March to July, respectively. The median value of *G*_*p*_ values was used to represent a seasonal suitability index *G*_*s*_ for a season *s* as follows:

11 $${G}_{s}={\rm{{\rm A}}}({\bf{G}}),$$

where ***G*** = {*G*_*p*_|*p* = 1, 2, …, *n*_*p*_}. *A* represents a function to determine a median value. *A* was used to determine a central tendency of climate conditions for the grass species in a season when the length of a growing period was unknown.

A growing season would start from a planting date. Still, planting date is often unknown, especially in regions where a grass species is not cultivated. When no planting date was known for the sites of interest, the planting date was set to be the first day of each season. For example, the planting date was assumed to be the first day of the first month for an individual growing season when the climate suitability index was determined using monthly data. The final suitability index *G* at a site was chosen to be the maximum value of *G*_*s*_ as follows:

12 $$G=\,\max \,\{{G}_{s}|s=1,2,\ldots ,\,{\rm{n}}\}$$

where *n* is the number of potential seasons. When no specific season was defined, the *G*_*s*_ values were determined for every potential season. For example, *G*_*s*_ was calculated for 12 seasons that start each month in a year (*n* = 12). Customized software written in C++ was used to determine the value of *G*.

### The parameter values for annual ryegrass based on existing knowledge

The climate suitability index for annual ryegrass was determined using the OR_F_ and AND_F_ models. Klir & Yuan^37^ suggested that an expert’s knowledge or existing data would be useful to determine parameters of a membership function. In the present study, the parameters of membership functions for annual ryegrass were determined using the FAO-EcoCrop database (Table 2). Because the EcoCrop database provides the range of annual precipitation, these parameter values were divided by 12 months for the model based on fuzzy logic. It was assumed that even distribution of rainfall across months would represent monthly climate envelopes for growth of a grass species. The lethal temperature for annual ryegrass from the EcoCrop database was −4 °C, which is relatively higher than that in previous reports. For example, Eagles *et al*.^42^ reported that a lethal temperature for annual ryegrass was −10 °C after a week of hardening. Because climate sensibility index would be calculated for prolonged winter chill conditions rather than short-lived winter spells, it was assumed that annual ryegrass would go through hardening for low temperature. Thus, the lethal temperature was set to be −10 °C. These parameter values were used for models based on fuzzy logic systems and the EcoCrop model to determine climate suitablity index for annual ryegrass.

**Table 2:** The model parameters to assess the climate suitability of forage crops including annual ryegrass, alfalfa, and sorghum obtained from existing knowledge.

Name	Description	Annual Ryegrass^b^	Alfalfa^b^	Sorghum^b^
G_min_	minimum growing period (d)	90	100	90
G_max_	maximum growing period (d)	270	210	300
T_kill_	lethal temperature (°C)	−10^c^	−25	0
T_min_	minimum absolute temperature (°C)	2	5	8
T_max_	maximum absolute temperature (°C)	38	45	40
T_OPmin_	minimum optimal temperature (°C)	14	21	27
T_OPmax_	maximum optimal temperature (^o^C)	30	27	35
R_min_^a^	minimum absolute rainfall (mm)	200	350	300
R_max_^a^	maximum absolute rainfall (mm)	1800	2700	3000
R_OPmin_^a^	minimum optimal rainfall (mm)	500	600	500
R_OPmax_^a^	maximum optimal rainfall (mm)	900	1200	1000

^a^The values of precipitation parameters were divided by 12 for the models based on fuzzy sets to represent monthly precipitation.

^b^The EcoCrop database operated by the Food and Agriculture Organization (FAO) was used to determine the parameter values of each crop.

^c^The lethal temperature was obtained from Eagles *et al*.^42^.

### Evaluation of climate suitability index using yield of annual ryegrass at site-years

The determination coefficient between climate suitability index and yield of annual ryegrass was calculated to examine if the model based on the fuzzy logic system would have a reasonable relationship between the climate suitability index and yield. Yield of annual ryegrass was obtained for 18 sites in three countries, including the USA, Belgium, and Australia (Appendix C).

Daily weather data were compiled to prepare inputs to the climate suitability models (Table 3). Average of temperature and sum of precipitation for a 30-day period were used as inputs for the models. These summary data were obtained for the duration of growing period (Appendix D). For the EcoCrop model, the sum of precipitation for the 12 consecutive 30-day periods was obtained because the model requires annual precipitation as an input. Instead of the first day of a calendar month, a planting date at a site-year was set to be the first day of the given season. Such data would minimize the impact of the uncertainty associated with climate inputs to the models during the season.

**Table 3:** List of weather stations for which weather data were obtained.

Name	Network^b^	ID^c^	Lat^d^	Long^d^
Mutdapilly	BOM	040004	−27.63	152.70
Gatton	BOM	040436	−27.55	152.33
Munte	GSOD	64280	50.93	3.67
Lexington^a^	GHCN:AWOS	USW00093820	38.04	−84.61
Franklinton	GHCN:AWOS	USW00093919	31.18	−90.47
Jeanerette^a^	GHCN:COOP	USC00164674	29.96	−91.71
Rosepine	GHCN:COOP	USC00162367	30.84	−93.29
Winnsboro^a^	GHCN:COOP	USC00169806	32.10	−91.70
Newton	GHCN:COOP	USC00226308	32.34	−89.08
Starkville	GHCN:COOP	USC00228374	33.47	−88.78
Holly Springs	GHCN:COOP	USC00224173	34.82	−89.43
Poplarville	GHCN:COOP	USC00227128	30.84	−89.55
Raymond	GHCN:COOP	USC00226476	32.21	−90.51
Ardmore^a^	GHCN:COOP	USC00340292	34.18	−97.18
Overton	GHCN:AWOS	USW00003901	32.38	−94.71
College Station	GHCN:AWOS	USW00003904	30.59	−96.36
Beaumont	GHCN:COOP	USC00410611	30.10	−94.10
Blackstone	GHCN:COOP	USC00441322	37.04	−77.94

^a^Missing data were replaced by weather observations at alternative weather stations for Lexington (GSOD; 724220), Ardmore (GHCN:COOP; USC00340312), Jeanerette (GHCN:AWOS; USW00053915), and Winnsboro (GHCN:COOP; USC00169804).

^b^BOM, GSOD, GHCN, AWOS, COOP indicate the Bureau of Meteorology, Australia, Global Surface Summary Of the Day, Global Historical Climatology Network, Automated Weather Observing System, and Cooperative Observer Network, respectively.

^c^The identification code for the corresponding weather station network.

^d^Lat and Long represent latitude and longitude, respectively.

### Spatial analysis of climate suitability index for annual ryegrass

The spatial distribution of the climate suitability index was also determined. Monthly climate surfaces were used as inputs to the models to generate maps of climate suitability index. The climatic data reported by Fick and Hijmans^43^ were obtained from the worldclim website (http://www.worldclim.org). Climate surfaces at a 1 km spatial resolution were used as inputs to the climate suitability models. Sowing dates for annual ryegrass was unknown for most of the grid cells. Thus, the climate suitablity index for each grid cell was determined using Eq. 12.

A likelihood analysis was performed to compare reliability of climate suitability index obtained from each model using the occurrence sites where annual ryegrass has been reported. The occurrence data of annual ryegrass were obtained from the Global Biodiversity Information Facility (GBIF) database (http://www.gbif.org). In total, 27,000 sites were compiled after eliminating sites without geographic coordinates. The occurrence site was denoted by *y* *=* 1. The probability that annual ryegrass would occur at a site was denoted by *Ψ(y* = *1)*. The probability of occurrence for a given climate suitability index *Ψ(y* = *1|G)* can be computed using Bayes rule as follows^44^:

13 $$\psi (y=1|G)=\pi (G|y=1)\cdot \psi (y=1)/\pi (G),$$

where *π(G|y* = *1)* indicates the conditional probability distribution of *G* at occurrence sites. It was assumed that *π*(*G|y* = *1)* would be similar to the probability distribution of *G* at occurrence sites obtained from the GBIF database. *π(G)* is the probability distribution function of *G* over a region.

The likelihood of occurrence for a given climate suitability index *G*_*i*_ would vary. The joint likelihood function *Λ* can be defined as follows:

14 $$\Lambda =\psi (y=1)\cdot L,$$

where *L* is *Π π(*G_*i*_*|y*_*i*_ = *1)/π*(*G*_*i*_*)*. The value of *Λ* for a given model differs by *L* because *Ψ(y* = *1)* would be constant for a given species. The smaller value of the negative logarithm of *L* results in the greater value of *Λ*. Thus, the model that has the smallest value of –log(*L*) would have the greatest reliability, e.g., the greatest likelihood of occurrence for a given index, compared with other models. The values of *L* for the OR_F_, AND_F_, and EcoCrop models were denoted by *L*_*OR*_, *L*_*AND*_, and *L*_*ECO*_, respectively.

The empirical cumulative distribution function (ECDF) of climate suitability index was obtained for each model. The ECDF of climate suitability index in grid cells corresponding to the occurrence sites *E*_*O*_ was determined to calculate *π(*G_*i*_*|y*_*i*_ = *1*). Another ECDF of the index for all grid cells *E*_*R*_ were also detetermined to calculate *π(G*_*i*_*)*. R, which is the open source statistical package^45^, was used to obtain these ECDFs. The value of *L* was estimated as follows:

15 $$L={E}_{o}({G}_{0})/{E}_{R}({G}_{0})\cdot {\prod }_{i=1}^{100}[{E}_{o}({G}_{i})-{E}_{o}({G}_{i-1})]/[{E}_{R}({G}_{i})-{E}_{R}({G}_{i-1})],$$

where *G*_*i*_ = 0.01· *i*.

### Spatial analysis of the climate suitability index for alfalfa and sorghum

The fuzzy logic system was applied to alfalfa and sorghum to examine if the fuzzy logic system would be applicable to other crops. The parameters of the fuzzy logic systems for both crops were obtained from the EcoCrop database (Table 2). The parameter values of the EcoCrop model for alfalfa were obtained from the EcoCrop database, whereas those for sorghum were determined using Ramirez-Villegas *et al*.^33^. A combined suitablity index of the EcoCrop model was calculated for sorghum as Ramirez-Villegas *et al*.^33^ suggested. The suitability index for alfalfa and sorghum was calculated at a global scale using the same gridded climate data. In total, 31,000 occurrence sites for alfalfa and 8,800 for sorghum were obtained from the GBIF database. The ECDF of climate suitability index at the occurrence sites was determined using maps of the climate suitablity index at a 1 km spatial resolution. The ECDF was used to compare the likelihood of occurrence for climate suitability index of the EcoCrop model and the model based on the fuzzy logic system. Results datasets are accessible from the following link: https://figshare.com/articles/climate_suitablity_of_annual_ryegrass_alfalfa_and_sorghum/5500876, (10.6084/m9.figshare.5500876)

## Results

### Comparison between climate suitability index and yield of annual ryegrass

**Figure 3:**
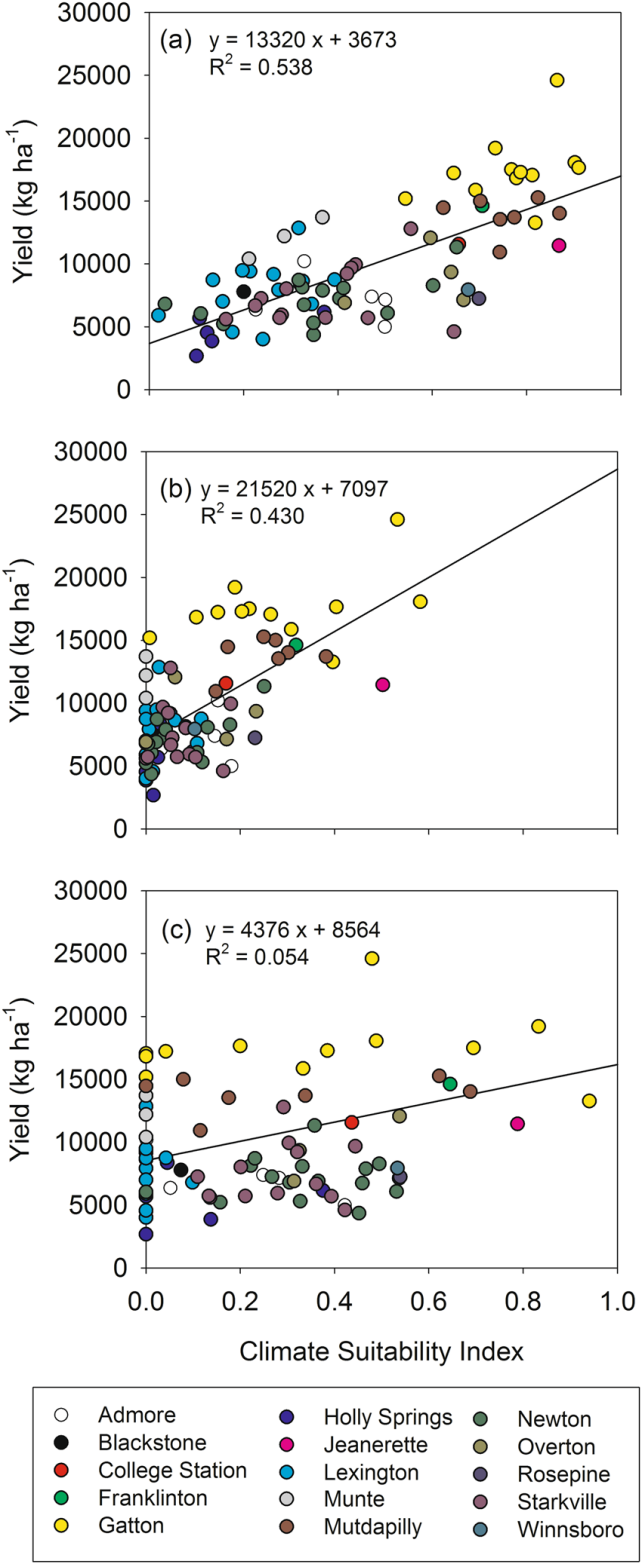
Distribution of observed yield at site-year for climate suitability index values of the OR_F_ model (**a**), the AND_F_ model (**b**), and the EcoCrop model (**c**), respectively. A line in each plot represents a regression line between yield and the climate suitability index of the corresponding model.

The climate suitability index of the OR_F_ model had the largest coefficient of determination for yield among the three models (Fig. 3). For example, the OR_F_ model accounted for 54% of the explained variation in yield for the 84 site-years except for sites where disease occurrence has been reported (Appendix C). The values of the climate suitability index were relatively low for site-years where low yield was observed. In contrast, the greater values of the climate suitability index did not necessarily coincide with high yield, which created a yield distribution similar to a triangular shape.

The AND_F_ and EcoCrop models, which are based on t-norm, tended to have zero climate suitability index for site-years where a relatively large yield was observed. The climate suitability index of the AND_F_ model was zero at Munte where a high yield above 10,000 kg/ha was observed for all of the study periods. The EcoCrop model had zero values of climate suitability index for more than 50% of the site-years although the yield ranged from 2,672 to 17,055 kg ha^−1^ for the corresponding periods. As a result, the coefficient of determination between the climate suitability index and yield at the site-years was considerably low (0.05) for the EcoCrop model compared to the other models.

### Spatial distribution of the climate suitability index

**Figure 4:**
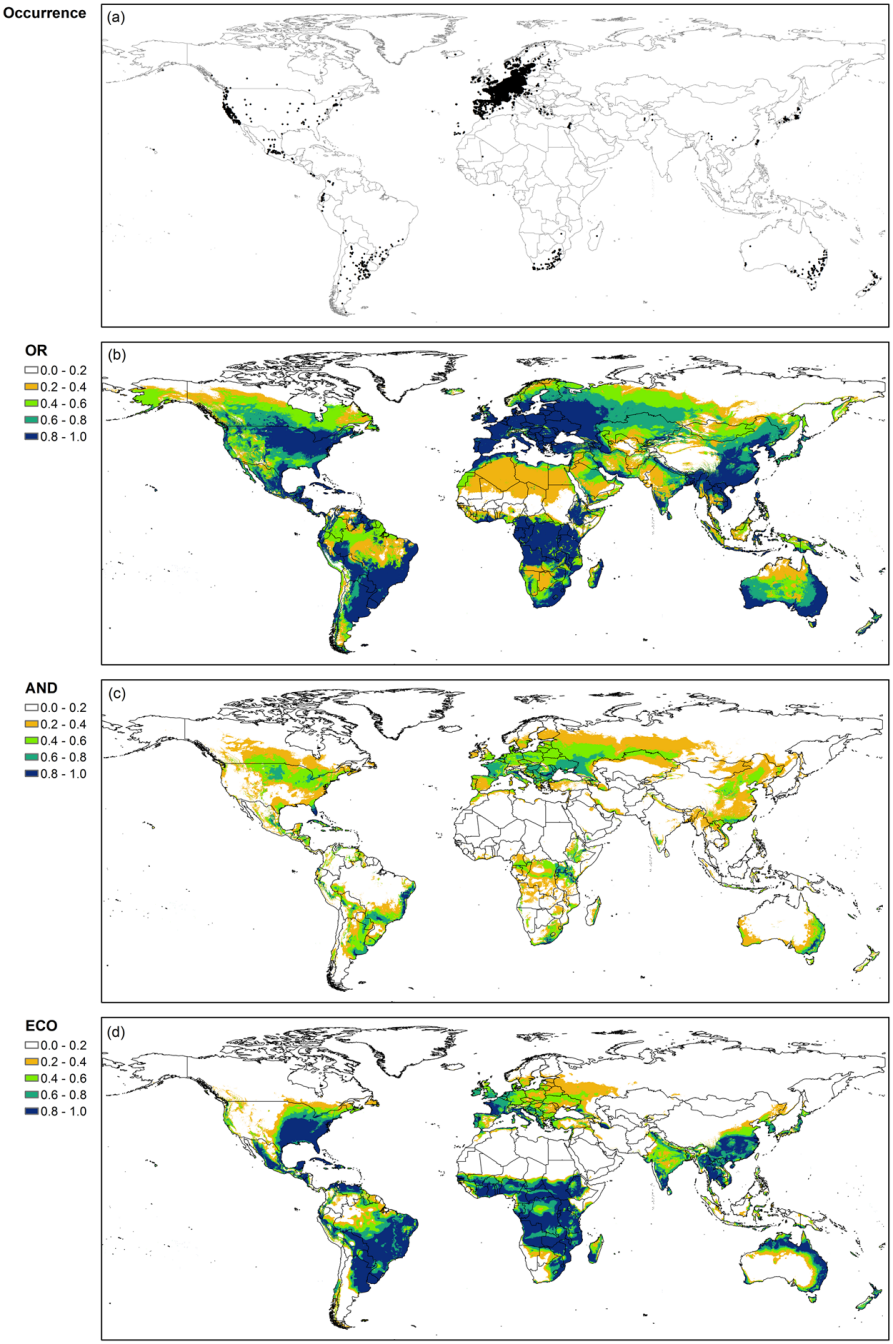
Map of global occurrence (**a**) and climate suitability index for annual ryegrass (**b–d**) obtained from the OR_F_ model (OR; **b**), the AND_F_ model (AND; **c**), and the EcoCrop model (ECO; **d**), respectively. ArcMap (version 10.0; http://desktop.arcgis.com/en/arcmap/) was used to create the maps.

**Figure 5:**
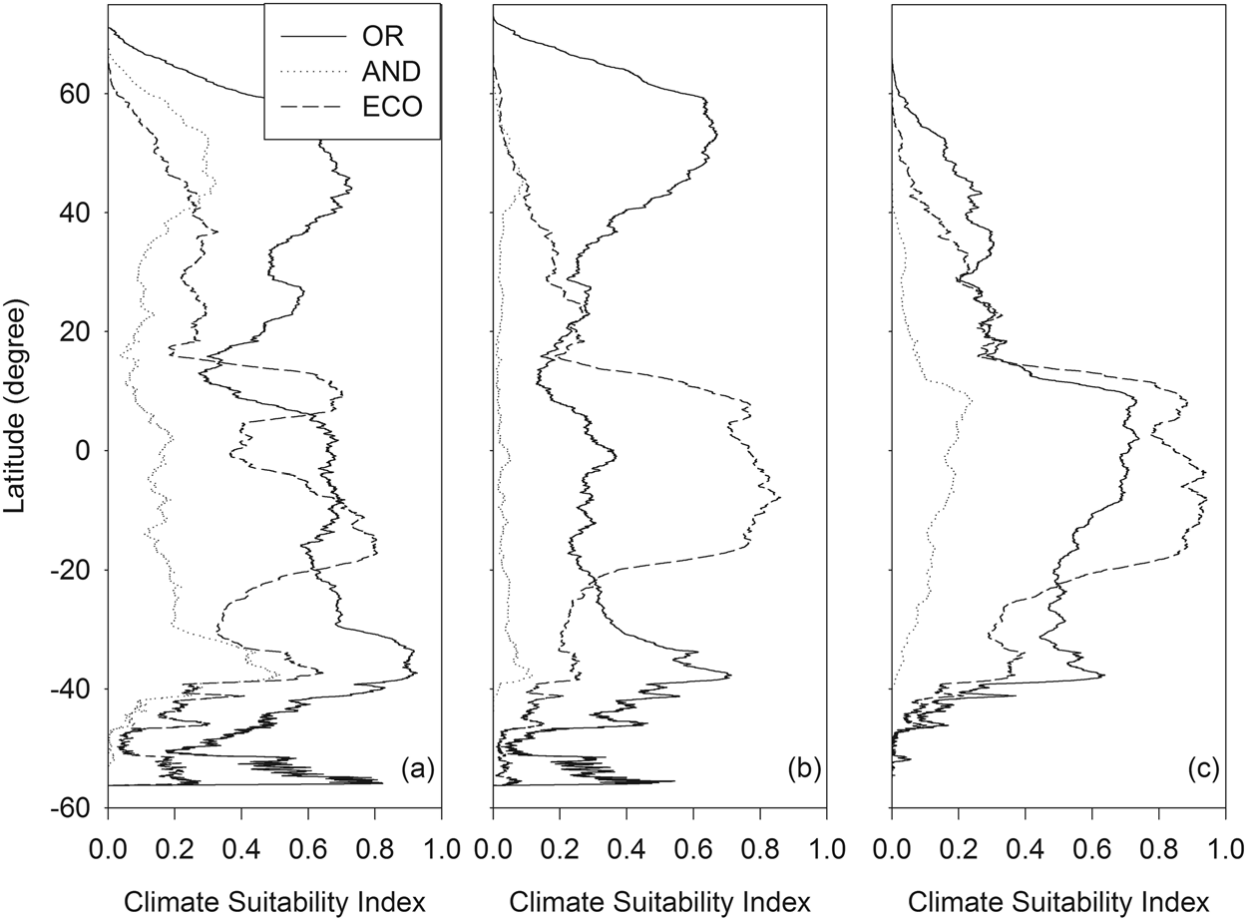
Average climate suitability index of annual ryegrass (**a**), alfalfa (**b**), and sorghum (**c**) by latitude. The values of climate suitability index were obtained from the OR_F_ model (OR), the AND_F_ model (AND), and the EcoCrop model (ECO), respectively.

Both OR_F_ and AND_F_ models tended to have a greater suitability index for mid and high latitude areas where annual ryegrass grows readily (Figs 4 and 5a). For example, the average climate suitability index of the OR_F_ model was >0.6 between 35° and 55°. Spatial distribution of climate suitability index of the AND_F_ model was similar to that of the OR_F_ model although the magnitude of the index for the AND_F_ model was smaller than that for OR_F_ model. In contrast, the EcoCrop model tended to have a greater climate suitability index for low latitude areas, e.g., between −20° and 20°, than for mid-high latitude areas.

**Figure 6:**
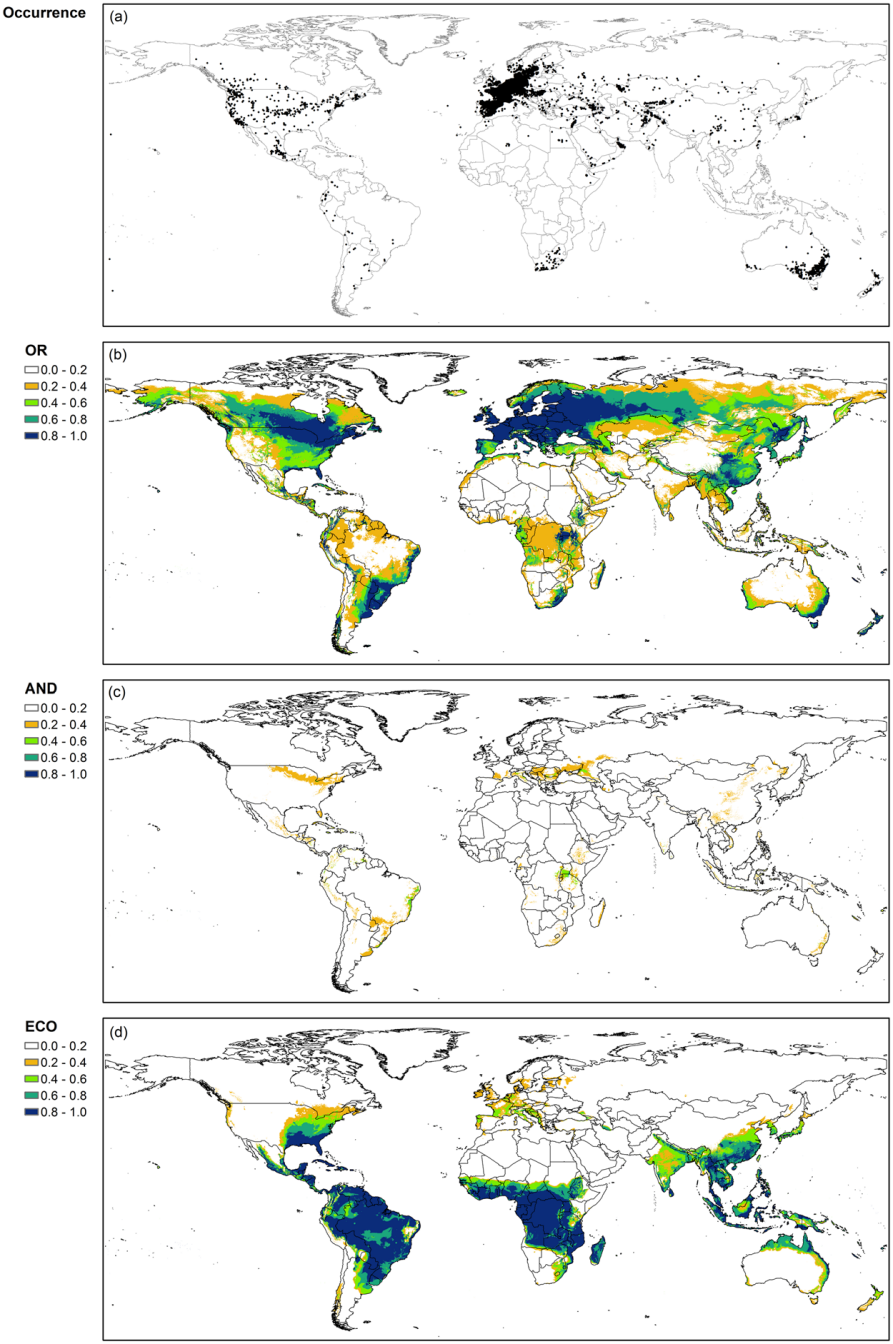
Map of global occurrence (**a**) and climate suitability index for alfalfa (**b–d**) obtained from the OR_F_ model (OR; **b**), the AND_F_ model (AND; **c**), and the EcoCrop model (ECO; **d**), respectively. ArcMap (version 10.0; http://desktop.arcgis.com/en/arcmap/) was used to create the maps.

The climate suitability index of the OR_F_ model for alfalfa was relatively high for mid latitude areas where the most occurrence sites were located (Figs 5b and 6). Areas with a high suitability index, e.g., >0.90, were large in Europe and the northern part of North America. The values of climate suitability index >0.90 were obtained only for small areas in Brazil and Africa. Similarly, the high values of the index for the AND_F_ model tended to coincide with sites where alfalfa has been grown. On the other hand, the EcoCrop model had considerably high values for the suitability index for the southern hemisphere where only a small number of occurrence sites have been recorded.

**Figure 7:**
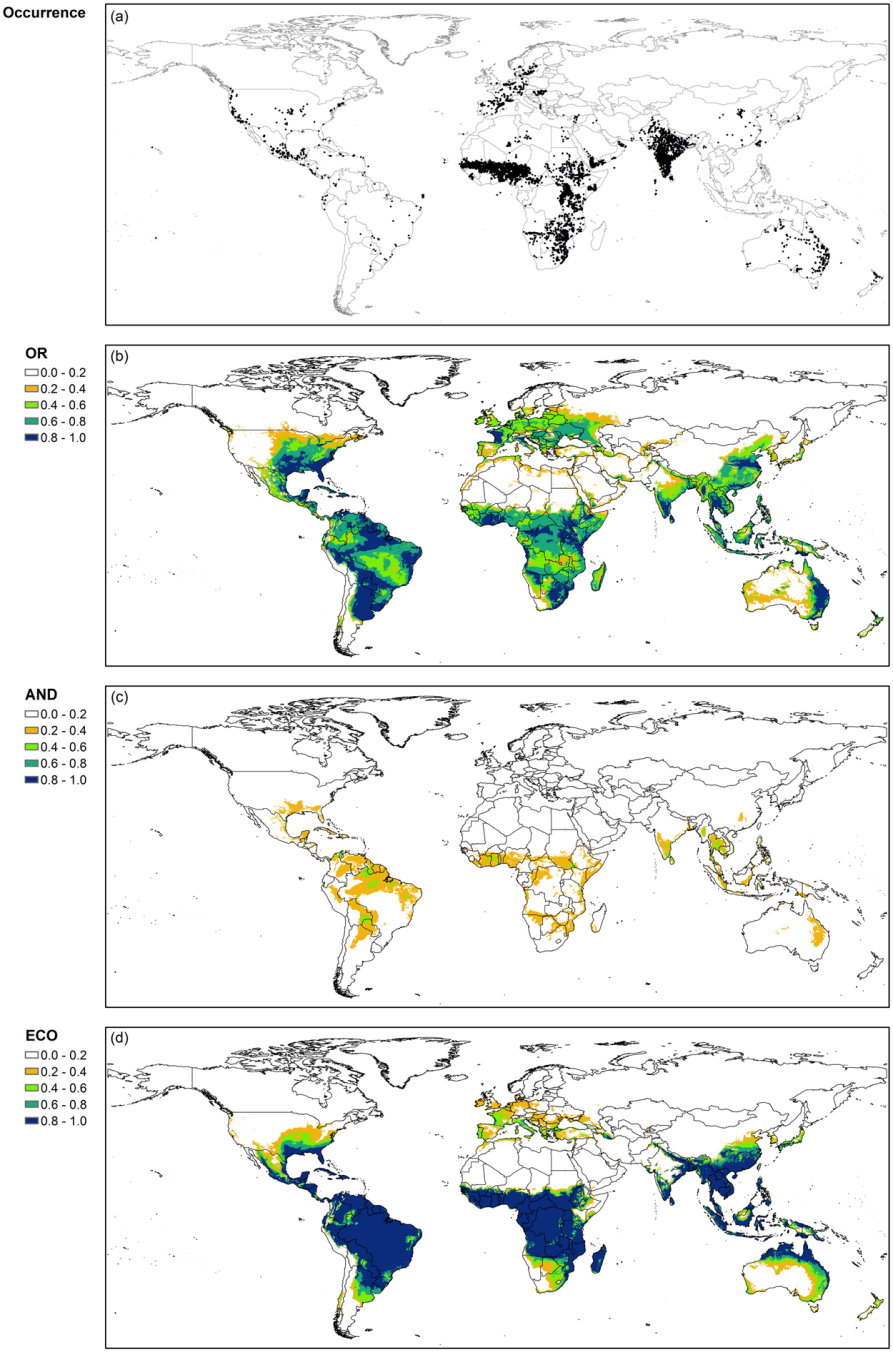
Map of global occurrence (**a**) and climate suitability index for sorghum (**b–d**) obtained from the OR_F_ model (OR; **b**), the AND_F_ model (AND; **c**), and the EcoCrop model (ECO; **d**), respectively. ArcMap (version 10.0; http://desktop.arcgis.com/en/arcmap/) was used to create the maps.

The spatial distribution of climate suitability indices for both OR_F_ and AND_F_ models differed between alfalfa and sorghum, whereas that for the EcoCrop model was relatively similar for both crops. The OR_F_ model had relatively greater values of climate suitability index for sorghum in low latitude areas where the most occurrence sites located (Figs 5–7). The AND_F_ model had a similar distribution of climate suitability index over latitude with the OR_F_ model although the magnitude of climate suitability index was considerably smaller for the AND_F_ model than the OR_F_ model. The EcoCrop model also tended to have a relatively similar distribution of climate suitability index for sorghum with the OR_F_ model and AND_F_ model, although the climate suitability index was relatively low for the northern Europe.

The OR_F_ model had a climate suitability index >0 at occurrence sites for alfalfa and sorghum (Figs 6 and 7). In contrast, the AND_F_ model and the EcoCrop models had zero values of climate suitability index at a considerably large number of occurrence sites. For example, the climate suitability index of the AND_F_ model was zero at about 51% of occurrence sites for alfalfa and 28% for sorghum. In addition, the climate suitability indices for both crops were relatively low mostly for areas with dry climate conditions where irrigation is usually performed, such as the northwestern US for alfalfa and northern India for sorghum.

**Figure 8:**
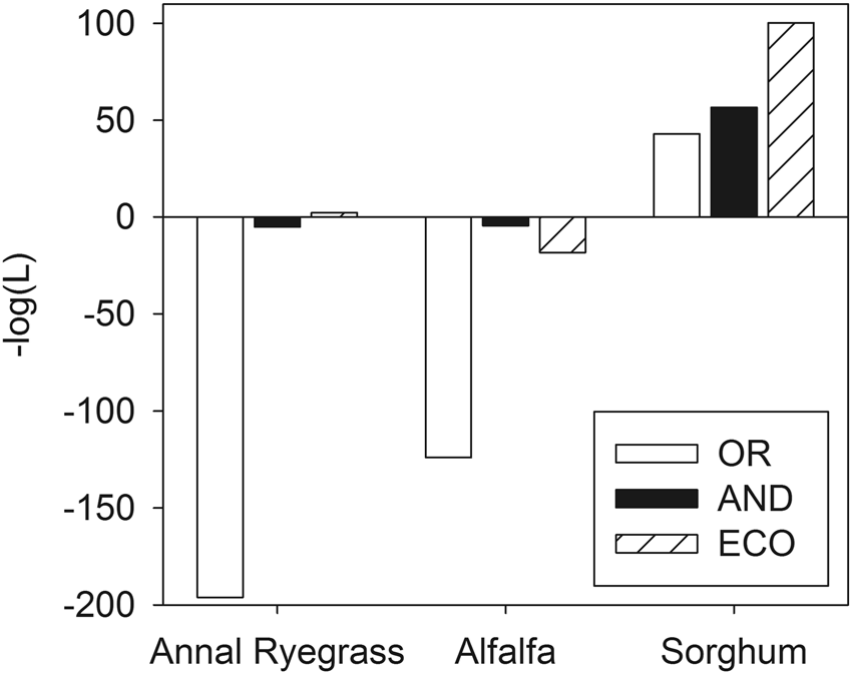
Comparison of the negative logarithm of *L* to maximize the joint likelihood of occurrence for a given climate suitability index. The climate suitability index for the models based on t-conorm and t-norm, and the EcoCrop model are denoted by *OR*, *AND*, and *ECO*, respectively.

The climate suitability index of the OR_F_ model had the greatest likelihood of occurrence for annual ryegrass, alfalfa, and sorghum of the three models (Fig. 8). For example, the value of –log(*L*_*OR*_) for annual ryegrass was −196 whereas those for

–log(*L*_*AND*_) and –log(*L*_*ECO*_) were −5 and 2, respectively. The negative logarithm of *L* tended to be smaller for annual ryegrass and alfalfa than that for sorghum for all models. The value of –log(*L*_*ECO*_) was smaller than that of –log(*L*_*AND*_) only for alfalfa.

## Discussion

This study demonstrated that the use of t-conorm such as the OR_F_ model was beneficial in classifying sites where climate quality would be suitable for growth of a crop. The OR_F_ model had the greatest likelihood of occurrence for the climate suitability index of the three models used in this study. The spatial distribution of climate suitability index for the OR_F_ model was similar to that of occurrence sites. The climate suitability index of the OR_F_ model was also highly correlated with yield of a crop, e.g., annual ryegrass, when weather data for the site were used. These findings suggest that the OR_F_ model based on t-conorm provides a more reliable assessment of climate suitability for the crops of interest compared to the other models based on the t-norm. The application of the t-conorm allowed for a reasonable representation of plant response to temperature and precipitation conditions in the assessment of climate suitability. The same rule statements and parameter values were applied to both OR_F_ and AND_F_ models, except for the logical operation between suitability of temperature and moisture. Still, the OR_F_ model, which is based on the t-conorm, had the most reliable assessment of climate suitability of three crops compared with the AND_F_ models based on the other logical operation, i.e., the t-norm. Thus, a choice of logical operation, e.g., t-conorm over t-norm, in assessment of climate quality for survival and growth of a plant would have a considerable impact on the reliability of a climate suitability index.

Our results indicated that incorporation of rules to represent climate conditions for growth of a species allowed reasonable assessment of climate suitability. Temperature and precipitation suitability of the fuzzy logic system was determined to take into account the response of a grass species to given climate conditions in terms of survival and growth. As a result, the climate suitability index based on the AND_F_ model had a greater correlation with yields of annual ryegrass than the one based on the EcoCrop model although the parameter values of both models were identical. Such observations would result from, in part, the fact that the EcoCrop model depended on climate variables that could explain a relatively small variation in growth of a species for the Law of the Minimum.

Although it is possible for a species to survive in an area where climate conditions are marginal, those marginal conditions would be the subset of conditions suitable for the species. Because a t-norm is used to determine an intersection of those conditions, it would be challenging to take into account climate conditions marginally suitable for growth of a species. As a result, a climate suitability index of zero was obtained even for sites where crop yield was considerably high when the t-norm was used to determine climate suitability index. Estes *et al*.^15^ reported that a species distribution model explained more variation in yield when the model was calibrated using sites with a high yield rather than all the occurrence sites. This previous finding could be related to those problems associated with a model based on t-norm.

Approaches based on a t-norm such as the AND_F_ model and the EcoCrop model, would suffer from collinearity between temperature and precipitation in relation to plant growth in assessing climate suitability of a species. Chakraborty *et al*.^46^ reported that temperature variables had a greater impact on growth performance than precipitation-related variables. Under high-temperature conditions, for example, the growth of annual ryegrass could benefit from ample soil moisture, which could result from optimum rainfall. Such an interaction between temperature and precipitation could not be realized using a t-norm.

The OR_F_ model would have limitation in assessing climate suitability of a crop due to its assumption such that a grower would manage a crop to maximize its yield under a rain-fed condition. Accurate assessment of climate suitability for a crop would require managements information such as planting date or irrigation options. However, such information is usually available only for major crops and not for other crops such as forages^47^. This would result in overprediction for the OR_F_ model because growers may be unable to select the best management option due to a lower priority for the forage crops or limited knowledge on climate. On the other hand, a crop can be grown in an irrigated area with low climate suitability, e.g., under a semi-arid climate condition. In such an area, it is likely that the OR_F_ model would underpredict the climate suitability index for a crop.

The OR_F_ model can be extended to improve assessment of climate suitability using other abiotic variables associated with crop productivity. For example, solar radiation has been used a required input variable for a process-based crop model in order to simulate photosynthesis and growth accurately^9^. Soil conditions can also play an important role in assessing crop suitability^34^. These variables can be incorporated into a climate suitability model when a large field data become available through an optimization process for the model. Benitez and Casillas^23^ and Fernandez *et al*.^24^ used an optimization approach to develop a hierarchical fuzzy model that has a similar architecture to the OR_F_ model. A hybrid approach including combination of fuzzy logic, clustering, and classification as well as soft computing can also be used to improve the OR_F_ model^48,49^. In addition, distribution of multiple species can be assessed using the fuzzy logic system that is derived from knowledge on interaction between multiple species as well as observation data^50^. A sensitivity analysis would be needed for further application of the OR_F_ model. The OR_F_ model can be used to support decision making for the design of a new cropping system in a region, which could be used for the development of climate change adaptation options^1^. For example, Rippke *et al*.^30^ showed that assessment of climate suitability for a crop would help with the development of climate change adaptation polices. Still, the uncertainty of the OR_F_ model needs to be analyzed before it is used in practice^51^. Janssen *et al*.^52^ suggested that the uncertainty of a fuzzy logic system can be assessed in terms of context, model structure, parameter, and input data. For example, a temporal resolution of climate input data could have a considerable impact on the uncertainty of the OR_F_ model (Appendix E). Thus, the framework for uncertainty analysis such as suggested by Walker *et al*.^53^ can be used for the OR_F_ model.

It would be preferable to examine the circumstances under which different types of logical operation would be better suited to represent the impact of temperature and rainfall conditions on climate suitability. For example, Genesis and Jonas^54^ used the Fuzzy Gamma overlay operations to predict potential yields of food and income crops. Zabel *et al*.^38^ assessed land suitability for the cultivation of various crops using a t-norm. Combination of different logical operations, e.g., t-conorm and gamma overlay function, could improve the reliability of the climate suitability assessment of a species. For example, an ensemble approach could be used for the assessment of climate suitability because a diverse set of methods has been developed for quantifying spatial distribution of a species^55^. Still, the OR_F_ model had advantages over other approaches because its parameters could be obtained from an existing knowledge recorded in the Ecocrop database and published literature.

The results from this study showed that a model based on t-conorm resulted in more reliable assessment of climate suitability for annual ryegrass, alfalfa, and sorghum compared with a model based on t-norm. This suggested that climate suitability assessment can be improved by taking into account the quality of environmental conditions for growth as well as survival of a species. In particular, the proposed algorithm in this study can provide an alternative approach to the Law of the Minimum that has been used to determine spatial distribution of a species. Our results also suggested that climate suitability of a species can be assessed without an iterative calibration process using existing knowledge, which could facilitate the impact assessment of biotic interaction on species distribution.

## Electronic supplementary material

supplementary info
