# Supplementary material for: Fuzzy Union to Assess Climate Suitability of Annual Ryegrass (Lolium multiflorum), Alfalfa (Medicago sativa) and Sorghum (Sorghum bicolor)
Source: Sci Rep. 2018 Jul 5;8:10220. doi: 10.1038/s41598-018-28291-3 (PMC6033868; doi:10.1038/s41598-018-28291-3)
Supplement: Supplementary file 1 — supplementary info [file 41598_2018_28291_MOESM1_ESM.docx]

**Fuzzy Union to Assess Climate Suitability of Annual Ryegrass (*Lolium multiflorum*), Alfalfa (*Medicago sativa*) and Sorghum (*Sorghum bicolor*)**

Hyunae Kim, Shin Woo Hyun, Gerrit Hoogenboom, Cheryl H. Porter, Kwang Soo Kim

Supplementary Information

Appendix A. The EcoCrop model

The EcoCrop model that determines suitability index for both temperature and precipitation has been used to identify areas suitable for a crop ^5^. Suitability of each variable could be interpreted as the degree to which the rule statements “temperature is suitable” and “rainfall is suitable” were met, respectively. Temperature and moisture conditions for a given crop are evaluated using monthly temperatures and annual precipitation.

The temperature suitability index of the EcoCrop model is based on monthly average temperature. A trapezoid function is used to represent the degree of temperature suitability as follows (Fig. 1a):

$T_{eco}(M_{m})=\left\{ \begin{matrix} \begin{matrix} \begin{matrix} 0 & & ,M_{m}< T_{min} \end{matrix} \\ \begin{matrix} \frac{T_{min}}{T_{min} - T_{OPmin}}+ \frac{M_{m}}{T_{OPmin}- T_{min}} & {, T}_{min}< M_{m}< T_{OPmin} & \end{matrix} \end{matrix} \\ \begin{matrix} \begin{matrix} 1 & & {,T}_{OPmin}< M_{m}< T_{OPmax} \end{matrix} \\ \begin{matrix} \frac{T_{max}}{T_{OPmax} - T_{max}}+ \frac{M_{m}}{T_{max}- T_{OPmax}} & {, T}_{OPmax}< M_{m} & \end{matrix} \end{matrix} \end{matrix} \right.$(Eq. A1),

where *T_eco_* and *M_m_* are the temperature suitability index function of the EcoCrop model and mean temperature in the month *m*. *T_min_*, *T_OPmin_*, *T_OPmax_*, and *T_max_* represent the parameters of the membership function for temperature suitability.

Suitability of precipitation is determined to take into account moisture conditions favorable for survival of a species. A degree of truth to the statement of rainfall condition, “precipitation is suitable” was determined using a trapezoid function as follows (Fig. 1b):

$P_{suitable}(P_{m})=\left\{ \begin{matrix} \begin{matrix} \begin{matrix} 0 & & , P_{m}<P_{min} or P_{m}>P_{max} \end{matrix} \\ \begin{matrix} \frac{{P_{m}- P}_{min}}{{P_{OPmin}- P}_{min}} & , P_{m}<P_{OPmin} & \end{matrix} \end{matrix} \\ \begin{matrix} 1 \begin{matrix} & & , P_{OPmin}<P_{m}< P_{OPmax} \end{matrix} \\ \begin{matrix} \frac{P_{max}-P_{m}}{{P_{max}- P}_{OPmax}} & {, P}_{m}<P_{OPmax} & \end{matrix} \end{matrix} \end{matrix} \right.$(Eq. A2),

where *P_m_* indicates monthly rainfall. *R_min_* and *R_max_* are the minimum and maximum range of rainfall for crop growth, respectively. Similarly, *R_OPmin_* and *R_OPmax_* represent the minimum and maximum range of rainfall for optimal crop growth, respectively. The annual precipitation was used as input to the membership function for the EcoCrop model.

Once the values of temperature suitability index are determined for each month of a given season, the seasonal temperature suitability index is obtained based on the minum of temperature suitability. The suitability of precipitation is multiplied by the seasonal temperature suitability to determine the final suitability index. The EcoCrop model also determines zero suitability under an extreme temperature condition, e.g., killing temperature, which would result in a crop failure. The EcoCrop model determines the climate suitability as the maximum of seasonal suitability under the assumption that planting date is unknown. To calculate climate suitability of the EcoCrop model, “dismo” package for R, which is an open source statistics analysis package, was used ^2^.

Appendix B. Definition of fuzzy sets

The fuzzy set for temperature suitability was defined using the duration of hours during which the optimal temperature conditions were met. The climate suitability models often evaluate the quality of temperature conditions using monthly temperatures, e.g., average, maximum, or minimum temperatures. When individual temperatures are used, fuzzy sets for corresponding temperatures would be need. Instead, the duration of temperature conditions during which crop growth would be suitable was used to determine the temperature suitablity in a given month. The value of *h_R_*, which is the number of hours during which the hourly temperature was within the range of optimum temperatures *R*, was used to assess temperature conditions. Temperature suitability would increase with an increasing value of *h_R_*. The fuzzy set that represents the suitability of temperature *T_suitable_* was defined as follows (Fig. 2a):

$T_{suitable}(X_{m},N_{m})=\left\{ \begin{matrix} 0 , X_{m}<T_{OPmin} or N_{m}> T_{OPmax} \\ \frac{h_{R}\left( X_{m},N_{m} \right)}{24} ,X_{m}\geq T_{OPmin} andN_{m}\leq T_{OPmax} \end{matrix} \right.$(Eq. B1),

where *X_m_* and *N_m_* indicate maximum and minimum temperature in a month *m*. *R* denotes [*T_OPmin_*, *T_OPmax_*]*.*

The value of *h_R_* was estimated using the sawtooth approach ^7^ (Fig. B1). The sawtooth method estimates the hourly temperature from daily data using linear interpolation between maximum and minimum temperature. Reicosky et al. ^7^ reported that the sawtooth model had an error equivalent to other methods that depend on more complex approaches, e.g., the use of a sinusoidal curve. It was assumed that monthly temperatures, e.g., averages of maximum and minimum temperature during a 30 day period, would represent typical daily temperatures in the given period. It was also assumed that the minimum temperature during the period would be identical for two consecutive days. When the monthly maximum temperature, *X_m_*, in a month *m* was less than the minimum value of the optimum temperature range, *T_OPmin_*, the value of *h_R_* for the range from *T*_OPmin_ to *T*_OPmax_ was set to be 0. Likewise, for the period during which the minimum temperature, *N_m_*, was greater than the maximum value of the optimum temperature range, *T_OPmax_*, the value of *h_R_* was also set to be 0. Otherwise, the value of *h_R_* was determined as follows (Fig. B1):

$h_{R}(X_{m}, N_{m})=\left[ \frac{Delta\left( X_{m}, max(N_{m},T_{OPmin} \right))}{tan\theta}-\frac{Delta\left( X_{m},T_{OPmax} \right)}{tan\theta} \right]\cdot2$ (Eq. B2),

where *Delta* represents the function that determines differences between *X_m_* and a lower temperature bound, e.g., *T_OPmax_* or *T_OPmin_*, as follows:

$Delta\left( a, b \right)= max(a-b, 0)$ (Eq. B3).

* * indicates the angle of a right triangle formed between hour and temperature in a day.

The value of *tanθ* was calculated as the ratio of the temperature range, i.e., *X_m_* – *N_m_*, to *t_adj_*, which is the duration of hours from the time of *N_m_* to the time of *X_m_* in a day (Fig. B1a). Although the value of *t_adj_* would differ by season and site, it was assumed to be 12 h, which would make it simple to calculate *h_R_* because *tanθ* becomes identical before and after the time of *X_m_* (Fig. B1b).

Another set of temperature suitability *T_reasonable_* was defined to examine if temperature conditions were reasonable for establishment in an early season. The membership function of fuzzy set for early temperature suitability was defined as follows (Fig. 2b):

$T_{reasonable}(X_{m},N_{m})=\left\{ \begin{matrix} 0 & & ,h_{[T_{min}, T_{OPmax}]}\left( X_{m},N_{m} \right)=0 \\ 1 & & ,h_{[T_{min}, T_{OPmax}]}\left( X_{m},N_{m} \right)>0 \end{matrix} \right.$ (Eq. B4).

Moisture conditions suitable for a species was determined in combination with the degree to which a given temperature condition was favorable for growth of a species. Temperature conditions favorable for growth of a given crop were evaluated using the rule statement, “maximum temperature is favorable and minimum temperature is favorable”. The bivalent membership of favorable temperature conditions was determined to examine if a given species would grow readily under *N_m_* and *X_m_*, respectively. The membership function that represents the term “favorable” was defined for monthly maximum *X_fav_* and minimum *N_fav_* temperatures as follows (Figs. 2c-d):

$X_{favorable}(X_{m})=\left\{ \begin{matrix} \begin{matrix} 0 & {,X}_{m} \end{matrix}>T_{OPmax} \\ \begin{matrix} 1 & ,X_{m}\leq T_{OPmax} \end{matrix} \end{matrix} \right.$ (Eq. B5)

and

$N_{favorable}(N_{m})=\left\{ \begin{matrix} \begin{matrix} 0 & , \end{matrix}N_{m}< T_{min} \\ \begin{matrix} 1 & , N_{m}\geq T_{min} \end{matrix} \end{matrix} \right.$ (Eq. B6).

The degree of membership for linguistic terms associated with “stressful” was determined using maximum and minimum temperatures in a month. The membership function of the term “stressful” for maximum temperature was defined as follows (Fig. 2e):

$X_{stressful}(X_{m})=\left\{ \begin{matrix} \begin{matrix} max(1,\frac{X_{m}-T_{OPmax}}{T_{max}-T_{OPmax}}) & {,X}_{m} \end{matrix}>T_{OPmax} \\ \begin{matrix} 0 & ,X_{m}\leq T_{OPmax} \end{matrix} \end{matrix} \right.$(Eq. B7).

Similarly, the membership function of the term “stressful” for minimum temperature was defined as follows (Fig. 2f):

$N_{stressful}(N_{m})=\left\{ \begin{matrix} \begin{matrix} 0 & \begin{matrix} & {, N}_{m}>T_{min} \end{matrix} \end{matrix} \\ \begin{matrix} \begin{matrix} max(1,\frac{T_{min}-N_{m}}{T_{min}-T_{kill}}) & , N_{m}\leq T_{min} \end{matrix} & \end{matrix} \end{matrix} \right.$, (Eq. B8)

where *T_kill_* indicates the killing temperature for a given crop.

It is likely that a species fails to survive when an extreme temperature condition occurs. Membership functions, *X_hamful_* and *N_hamful_* were used to evaluate the rule statements “maximum temperature was harmful” and “minimum temperature was harmful,” respectively. *X_hamful_* and *N_hamful_* were defined as follows (Figs. 2g-h):

$X_{harmful}(X_{m})=\left\{ \begin{matrix} \begin{matrix} 1 & ,X_{m} \end{matrix}>T_{max} \\ \begin{matrix} 0 & ,X_{m}\leq T_{max} \end{matrix} \end{matrix} \right.$ (Eq. B9).

and

$N_{harmful}(N_{m})=\left\{ \begin{matrix} \begin{matrix} 1 & , \end{matrix} N_{m}< T_{kill} \\ \begin{matrix} 0 & , N_{m}\geq T_{kill} \end{matrix} \end{matrix} \right.$ (Eq. B10).

The bivalent value for occurrence of the extreme conditions was determined to evaluate whether or not a species could survive under a given condition.

Appendix C. yield data of annual ryegrass

Yield data for annual ryegrass were collected from the literatures (Table C1). In total, yield for 98 site-years were obtained. Annual yield data were available by variety from the literature for all the sites except for Munte, Belgium (Fig. C1). The yield for a given variety was averaged to compare with the values of climate suitability index by site-year.

The climate suitability index was determined under the assumption that no biotic stress , e.g., pests, diseases, and weeds, would occur at the site of interest. Thus, yield data at sites where a serious disease has been reported were excluded from the comparison between yields and climate suitability index. For example, crown rust (*Puccinia coronate*) is the most serious foliar disease of ryegrass species ^6,9^. White & Lemus ^10^ suggested that crown rust would occasionally occur at sites near the coastal regions in the US including Poplarville, MS and Beaumont, TX.

Climate suitability index were compared with the ratio of yield between resistant and susceptible cultivars to examine if a given site would have potential disease risks. Yield of a cultivar resistant to a disease would be greater than that of the susceptible ones at sites where the disease would cause problems. It was assumed that the risk of the disease would be low at a site where a relative yield of a resistant cultivar to susceptible one was < 1. White and Lemus ^10^ suggested that Jumbo had better resistance to crown rust than Marshall. Yield for both Jumbo and Marshall was also available at more site-years than any other cultivars. The climate suitability index using each model was compared with a relative yield of Jumbo to Marshall at site-years where yield for both cultivars had been reported for more than or equal to three years. The averages of relative yields for the extended periods, e.g., > three years, were compared with averages of climate suitability index by given sites.

Climate suitability index explained a considerable amount of variability in the relative yield of a resistant cultivar to susceptible one (Fig. C2). The R^2^ value of the EcoCrop model (0.725) was slightly greater than that of OR_F_ model (0.698). Although only six sites were available for the analysis, the p-values for correlation coefficients were < 0.05 for all of the models. The relative yield was >1 at Beaumont, Poplarville, and Raymond, which indicated a high risk of disease outbreak.

Appendix D. Management of daily weather data

The sites where daily weather data were obtained were listed in Table D1. Daily minimum and mean temperatures, and precipitation data were collected for sites in the US and Belgium from the Utah Climate Center (https://climate.usurf.usu.edu/). The Global Historical Climatology Network (GHCN) and Global Surface Summary of the Day (GSOD) were used to obtain data for the US and Belgium sites, respectively. For Australia sites, temperature and precipitation data were obtained from the Bureau of Meteorology, Australia (http://www.bom.gov.au/).

The gaps of temperature and precipitation were filled independently. When temperature data were missing for a short period, e.g., within two days, averages of temperature from adjacent days were used to fill the gap. Missing temperature data for two more consecutive days filled with average values for remaining data in a calendar month. When temperature or precipitation was missing for more than seven consecutive days in the month, missing temperature data were replaced by data from a nearby weather station. For missing precipitation data for less than 10 days in a month, it was assumed that no rainfall occurred. When the gap of precipitation was more than 10 days in a month, all of precipitation data were replaced by data from a nearby weather station.

Appendix E. Comparison of the duration of optimum temperature occurrence using daily and monthly data

The values of *h_[TOPMin, TOPMax]_* for annual ryegrass were obtained using monthly and daily temperatures. At first, the monthly values of *h_R_*, which is denoted by *h_R_*(month), were determined using monthly averages of daily maximum and minimum temperatures as inputs to eq.B2. Then, daily values of *h_R_*, which was calculated using daily temperatures as inputs to eq.B2, were averaged by month to determine another set of monthly *h_R_* values *h_R_*(day). Those values of *h_R_* were calculated using weather data at 98 sites-years. In addition, 71 site-year data, which are included in the Decision Support System for Agrotechnology transfer (DSSAT) package ^3^, were also used to represent sites with diverse climate conditions (Fig. F1). A clear linear relationship was found between *h_R_*(month) and *h_R_*(day) although the over-estimation error of *h_R_*(month) was pronounced for the greater values of *h_R_*(day). The value of *h_R_*(month) also tended to underestimate relatively small values of *h_R_*(day).

This result suggested that the reliability of the climate suitability index for the model based on a fuzzy logic system would be affected by temporal resolution of climate data. It is likely that the values of climate suitability index would be overestimated when monthly data would overestimate the value of *h_R_*. Temperature data under extreme conditions would occur less frequently in monthly summary of temperatures. Because global temperature surfaces are averages of monthly temperatures over a long time period, e.g., 30 years, frequency of extreme temperature would be much less than monthly temperatures at site years. As a result, the tendency of overestimation of *h_R_* would be amplified using those surface data.

Appendix F. Comparison of sensitivity, specificity, and true skill statistics

Sensitivity, specificity, and true skill statistic were determined to evaluate the predictive performance of the models based on fuzzy logic and the EcoCrop model (Table F1). Pseudo-absence sites were selected to represent locations where presence of a crop has not been reported. Maps of harvest area for mixed grassland, alfalfa, and sorghum were used to represent a presence area ^4^. It was assumed that each crop would be present in the area where harvested acreage was > 0. A buffer area within three degree (about 300 km) from the presence site was also used to assume presence area ^1^. The pseudo-absence sites were collected randomly from the areas outside the presence area. Because the outputs of models are between 0 and 1, a threshold value for each model to classify presence and absence of a crop was determined iteratively. Presence data and pseudo-absence data were pooled and grouped into five subsets. Then, five-fold cross validation was performed to determine sensitivity and specificity, which are the probability of true presence and true absence, respectively. True skill statistic (TSS) was determined as the difference between the sum of sensitivity and specificity and 1. The value of TSS for the OR_F_ model was > 0.69 for all the crops of interest whereas that for the other models was highly variable. However, the statistics based on pseudo-absence data should be used with caution. For example, a crop could be grown even in an area where climate suitability for the crop would be low when irrigation or other management practices could allow for its production. Thus, it is likely that specificity of the model would have a bias, which would result in the bias of TSS.

**REFERENCES**

1. Gomes, V.H.F. et al. Species Distribution Modelling: Contrasting presence-only models with plot abundance data. *Sci. Rep.* **8**. <https://doi.org/10.1038/s41598-017-18927-1> (2018)
2. Hijmans, R.J., Phillips, S., Leathwick, J. & Elith, J. Dismo: Species distribution modeling, R package version 1.0-15 <http://CRAN.R-project.org/package=dismo>, (accessed 01.11.2016) (2016).
3. Hoogenboom, G. et al. *Decision Support System for Agrotechnology Transfer (DSSAT) 2015, Version 4.6.1.* (DSSAT Foundation, 2015).
4. Monfreda, C., N. Ramankutty, and J.A. Foley. Farming the planet. Part 2: Geographic distribution of crop areas, yields, physiological types, and net primary production in the year 2000. *Global Biogeochem. Cycles* **22**, GB1022, doi:10.1029/2007GB002947 (2008).
5. Ramirez-Villegas, J., Jarvis, A. & Läderach, P. Empirical approaches for assessing impacts of climate change on agriculture: The EcoCrop model and a case study with grain sorghum. *Agr. Forest Meteorol.* **170**, 67-78 (2013).
6. Reheul, D. & Ghequiere, A. Breeding perennial ryegrass with better crown rust resistance. *Plant Breeding* **115**, 465-469 (1996).
7. Reicosky, D.C., Winkelman, L.J., Baker, J.M. & Baker, D.G. Accuracy of hourly air temperatures calculated from daily minima and maxima. *Agr. Forest Meteorol.* **46**, 193-209 (1989).
8. Sanders, C.G. Comments on the model for estimating the completion of rest for “Red Haven” and “Elberta” peach trees. *Hortic. Sci.* **10**, 560-561 (1975).
9. Takahashi, W. et al. Increased resistance to crown rust disease in transgenic Italian ryegrass (*Lolium multiflorum* Lam.) expressing the rice chitinase gene. *Plant Cell Rep.* **23**, 811-818 (2015).
10. White, J.A. & Lemus, R. Long-term summary of ryegrass varieties and ploidy types in Mississippi. *Am. J. Plant Sci.* **5**, 3151-3158 (2014).
11. Alison, W. et al. Performance of cool-season annual forage crops in Louisiana, 2007-2008. *LAES Research Summary No. 177.* (LSU Ag Center, 2007).
12. Edwards, N.C. et al. Mississippi forage crop variety trials, 2000. *Information Bulletin* **369** (Mississippi Agricultural & Forestry Experiment Station, 2000).
13. Edwards, N.C. et al. Mississippi forage crop variety trials, 2001. *Information Bulletin* **379** (Mississippi Agricultural & Forestry Experiment Station, 2001).
14. Guretzky, J.A. *Forage yield from 2006-2007 annual ryegrass variety trial* (The Samuel Roberts Noble Foundation, 2007).
15. Guretzky, J.A. *Forage yield from 2006-2007 annual ryegrass variety trial* (The Samuel Roberts Noble Foundation, 2008).
16. Lang, D. et al. Mississippi forage crop variety trials, 2002. *Information Bulletin* **379** (Mississippi Agricultural & Forestry Experiment Station, 2002).
17. Lang, D. et al. *Mississippi forage crop variety trials, 2003* (Mississippi Agricultural & Forestry Experiment Station, 2003).
18. Lang, D. et al. *Mississippi forage crop variety trials, 2004* (Mississippi Agricultural & Forestry Experiment Station, 2004).
19. Lang, D. & Johnson, B. mississippi ryegrass variety trials, 2004-2006. *Information Bulletin* **430** (Mississippi Agricultural & Forestry Experiment Station, 2006).
20. Lang, D., Johnson, B., Shankle, B. & Parish, J.R. Mississippi ryegrass variety trials, 2006-2008. *Information Bulletin* **450** (Mississippi Agricultural & Forestry Experiment Station, 2009).
21. Lemus, R. *Annual cool-season variety test report: Mississippi State Variety Testing Program, 2010-2011* (Mississippi Agricultural & Forestry Experiment Station, 2011).
22. Lowe, K.F. et al. Evaluating temperate species for the subtropics. 1. Annual ryegrass. *Trop. Grasslands* **41**, 9-25 (2007).
23. Mosali, J. *Forage yields from 2009-2010 ryegrass* (The Samuel Roberts Noble Foundation, 2010)
24. Mosali, J. *Forage yields from 2010-2011 ryegrass* (The Samuel Roberts Noble Foundation, 2011)
25. Mosali, J., Guretzky, J.A., Saha, M. & Norton, S. *Forage yields from 2008-2009 ryegrass* (The Samuel Roberts Noble Foundation, 2009).
26. Muylle, H. et al. Yield and energy balance of annual and perennial lignocellulosic crops for bio-refinery use: A 4-year field experiment in Belgium. *Eur. J. Agron.* **63**, 62-70 (2015).
27. Nelson, L.R., Crowder, J. & Rouquette, F.M. Registration of ‘Nelson’ annual ryegrass. *J. Plant Regist.* **5**, 1-4 (2011).
28. Olson, G.L. et al. *2004 Annual and perennial ryegrass report* (Kentucky Agricultural Experiment Station, University of Kentucky, 2005).
29. Olson, G.L., Smith, S.R., Philips, T.D. & Lacefield, G.D. *2006 Annual and perennial ryegrass report* (Kentucky Agricultural Experiment Station, University of Kentucky, 2007).
30. Olson, G.L., Smith, S.R., Philips, T.D. & Lacefield, G.D. *2007 Annual and perennial ryegrass report* (Kentucky Agricultural Experiment Station, University of Kentucky, 2008).
31. Olson, G.L., Smith, S.R., Philips, T.D. & Lacefield, G.D. *2008 Annual and perennial ryegrass report* (Kentucky Agricultural Experiment Station, University of Kentucky, 2009).
32. Olson, G.L., Smith, S.R., Philips, T.D. & Lacefield, G.D. *2009 Annual and perennial ryegrass and festulolium report* (Kentucky Agricultural Experiment Station, University of Kentucky, 2010).
33. Olson, G.L., Smith, S.R., Philips, T.D. & Lacefield, G.D. *2010 Annual and perennial ryegrass and festulolium report* (Kentucky Agricultural Experiment Station, University of Kentucky, 2011).
34. Olson, G.L., Smith, S.R., Philips, T.D. & Lacefield, G.D. *2011 Annual and perennial ryegrass and festulolium report* (Kentucky Agricultural Experiment Station, University of Kentucky, 2012).
35. Olson, G.L., Smith, S.R., Philips, T.D. & Lacefield, G.D. *2012 Annual and perennial ryegrass and festulolium report* (Kentucky Agricultural Experiment Station, University of Kentucky, 2013).
36. Olson, G.L., Smith, S.R., Philips, T.D. & Lacefield, G.D. *2013 Annual and perennial ryegrass and festulolium report* (Kentucky Agricultural Experiment Station, University of Kentucky, 2014).
37. Olson, G.L., Smith, S.R., Philips, T.D. & Lacefield, G.D. *2014 annual and perennial ryegrass and festulolium report* (Kentucky Agricultural Experiment Station, University of Kentucky, 2015).
38. Olson, G.L. et al. *2005 Annual and perennial ryegrass report* (Kentucky Agricultural Experiment Station, University of Kentucky, 2006).
39. Parish, J.R. Mississippi annual ryegrass variety trials, 2008-2009. *Information Bulletin* **45** (Mississippi Agricultural & Forestry Experiment Station, 2010).
40. Spitaleri, R.F., Collins, M., Lacefield, G.D. & Burris, P.B. *2003 Annual and perennial ryegrass report* (Kentucky Agricultural Experiment Station, University of Kentucky, 2004).
41. Spitaleri, R.F., Henning, J.C., Lacefield, G.D. & Burris, P.B. *2001 Annual and perennial ryegrass report* (Kentucky Agricultural Experiment Station, University of Kentucky, 2002).
42. Spitaleri, R.F., Henning, J.C., Lacefield, G.D. & Burris, P.B. *2002 Annual and perennial ryegrass report* (Kentucky Agricultural Experiment Station, University of Kentucky, 2003).
43. Teutsch, C.D. & Tilson, W.M. *2008 Annual ryegrass variety trial report* (Southern Piemont AREC, 2009).
44. Twidwell, E. et al. *2013-2014 Cool-season pasture and forage varieties. Pub. 2334.* (LSU Ag Center, 2013).
45. Twidwell, E. et al. *2014-2015 Cool-season pasture and forage varieties. Pub. 2334.* (LSU Ag Center, 2015).
46. White, J. et al. Mississippi annual cool-season forage crop, 2011-2012. *Information Bulletin* **470** (Mississippi Agricultural & Forestry Experiment Station, 2012).
47. White, J. et al. Mississippi annual cool-season forage crop, 2012-2013. *Information Bulletin* **479** (Mississippi Agricultural & Forestry Experiment Station, 2013).
48. White, J. et al. Mississippi annual cool-season forage crop, 2013-2014. *Information Bulletin* **488** (Mississippi Agricultural & Forestry Experiment Station, 2014).
49. White, J. et al. Mississippi annual cool-season forage crop variety trials, 2014-2015. *Information Bulletin* **501** (Mississippi Agricultural & Forestry Experiment Station, 2015).

**Table C1.** Sites and years for yield of annual ryegrass

| Location | Region^1^ | Year | References | Code^2^ |
| --- | --- | --- | --- | --- |
| Gatton | Australia | 1984-1987, 1989,  1991-1997 | Lowe et al. ^22^ | G |
| Mutdapilly | Australia | 1999-2005 | Lowe et al. ^22^ | U |
| Munte | Belgium | 2008-2010 | Muylle et al. ^26^ | M |
| Lexington | KY, USA | 1999-2013 | Spitaleri et al. ^40-42^  Olson et al. ^28-38^ | L |
| Franklinton | LA, USA | 2007, 2010-2014 | Alison et al. ^11^  Twidwell et al. ^44-45^ | F |
| Winnsboro | LA, USA | 2007, 2010-2014 | Alison et al. ^11^  Twidwell et al. ^44-45^ | W |
| Jeanerette | LA, USA | 2007, 2010-2014 | Alison et al. ^11^  Twidwell et al. ^44-45^ | J |
| Rosepine | LA, USA | 2007 | Alison et al. ^11^  Twidwell et al. ^44-45^ | E |
| Newton | MS, USA | 1999-2008, 2010-2014 | Edwards et al. ^12-13^  Lang et al. ^16-18,20^  Lang & Johnson ^19^  Parish ^39^, Lemus ^21^  White et al. ^46-49^ | N |
| Starkville | MS, USA | 2001-2008, 2010-2014 | Edwards et al. ^13^  Lang et al. ^16-18,20^  Lang & Johnson ^19^  Parish ^39^, Lemus ^21^  White et al. ^46-49^ | S |
| Poplarville | MS, USA | 1999, 2000, 2008, 2011-2014 | Edwards et al. ^12-13^  Lang et al. ^20^  Parish ^39^, Lemus ^21^  White et al. ^46-49^ | P |
| Holly springs | MS, USA | 2008, 2010-2014 | Lang et al. ^20^  Lemus ^21^  White et al. ^46-49^ | H |
| Raymond | MS, USA | 1999-2002 | Edwards et al. ^12-13^  Lang et al. ^16^ | R |
| Ardmore | OK, USA | 2006-2010 | Guretzky ^14-15^  Mosali et al. ^23-25^ | A |
| Overton | TX, USA | 2005-2007, 2009 | Nelson et al. ^27^ | O |
| Beaumont | TX, USA | 2005-2007 | Nelson et al. ^27^ | B |
| College Station | TX, USA | 2009 | Nelson et al. ^27^ | C |
| Blackstone | VA, USA | 2008 | Teutsch and Tilson ^43^ | K |

1. For the sites in the USA, KY, LA, MS, OK, TX, and VA indicates the states of Kentucky, Louisiana, Mississippi, Oklahoma, Texas, and Virginia states, respectively.
2. The code was used to indicate individual sites in figures.

**Table F1**. Comparison of statistics to assess the accuracy of models. Average and standard deviation of each statistic were obtained from five-fold cross validation using presence and pseudo-absence data.

| Statistics | Crop | OR^a^ |  | AND^a^ |  | ECO^a^ |
| --- | --- | --- | --- | --- | --- | --- |
| Sensitivity^b^ | Annual Ryegrass | 0.934 ±0.0087 |  | 0.915 ±0.0039 |  | 0.979 ±0.0050 |
|  | Alfalfa | 0.768 ±0.0019 |  | 0.458 ±0.0049 |  | 0.752 ±0.0048 |
|  | Sorghum | 0.884 ±0.0136 |  | 0.711 ±0.0127 |  | 0.862 ±0.0162 |
|  |  |  |  |  |  |  |
| Specificity^b^ | Annual Ryegrass | 0.863 ±0.0087 |  | 0.907 ±0.0039 |  | 0.739 ±0.0050 |
|  | Alfalfa | 0.928 ±0.0019 |  | 0.824 ±0.0049 |  | 0.663 ±0.0049 |
|  | Sorghum | 0.806 ±0.0136 |  | 0.858 ±0.0127 |  | 0.830 ±0.0162 |
|  |  |  |  |  |  |  |
| True Skill Statistic^c^ | Annual Ryegrass | 0.798 ±0.0047 |  | 0.823 ±0.0032 |  | 0.718 ±0.0030 |
|  | Alfalfa | 0.695 ±0.0064 |  | 0.279 ±0.0109 |  | 0.415 ±0.0072 |
|  | Sorghum | 0.693 ±0.0217 |  | 0.572 ±0.0195 |  | 0.694 ±0.0185 |

1. OR, AND, and ECO indicate the model based on the t-conorm, and the t-norm, and the EcoCrop model, respectively
2. Sensitivity and Specificity indicate the probabilities that a model will correctly classify a presence and absence, respectively.
3. True skill statistic = Sensitivity + Specificity - 1

Figure Captions

Figure B1. Illustration of determining the duration of hours, *h_R_*, during which a given range of temperature occurred: (a) when the sawtooth model is used to estimate hourly temperature using maximum *X_m_* and minimum *N_m_* temperatures in a day, the portion of *h_R_* would differ between morning and afternoon periods. The sawtooth model assumes that *X_m_* would occur at 15:00. (b) Under the assumption that *X_m_* would occur at 17:00 and *N_m_* is same for two consecutive days,  becomes identical for the periods before and after the time of *X_m_*, which makes it easy to determine *h_R_* using tan (b).­­­

Figure C2. The relationship between the relative yield of resistant cultivar to susceptible one and climate suitability index for (a) OR_F_ model, (b) AND_F_ model, and (c) EcoCrop model, respectively. the reference line indicate the relative yield become 1. Jumbo and Marshall were chosen to be resistant and susceptible cultivars to crown rust disease, respectively.

Figure E1. Sites where weather data were collected for data analysis. Climate suitability index was calculated for annual ryegrass at sites indicated by an upside-down triangle. Sites indicated by both triangles were used to compare the duration of hours within a range of temperatures calculated using daily and monthly temperatures. Weather data collected from the sites for assessment of climate suitability index in Australia, Belgium, and the US are denoted by Annual ryegrass. DSSAT indicate weather data included in the DSSAT (version 4.6). ArcMap (version 10.0; http://desktop.arcgis.com/en/arcmap/) was used to create the map.

Figure E2. Comparison of suitability of temperature calculated using daily and monthly temperatures as inputs to T_suitable_(X,N), which is the duration of hours *h_R_* within a range of temperatures. The range was defined from 14-30^o^C. X_m_ and N_m_ indicate monthly maximum and minimum temperature, respectively. Daily maximum and minimum temperatures are denoted by X_d_ and N_d_, respectively. n_d_ indicates the number of days in a month. Weather data collected from the sites for assessment of climate suitability index in Australia, Belgium, and the US are denoted by Annual ryegrass. DSSAT indicate weather data included in the DSSAT (version 4.6).

**Kim et al., Fig. B1.**

**Kim et al., Fig. C2.**


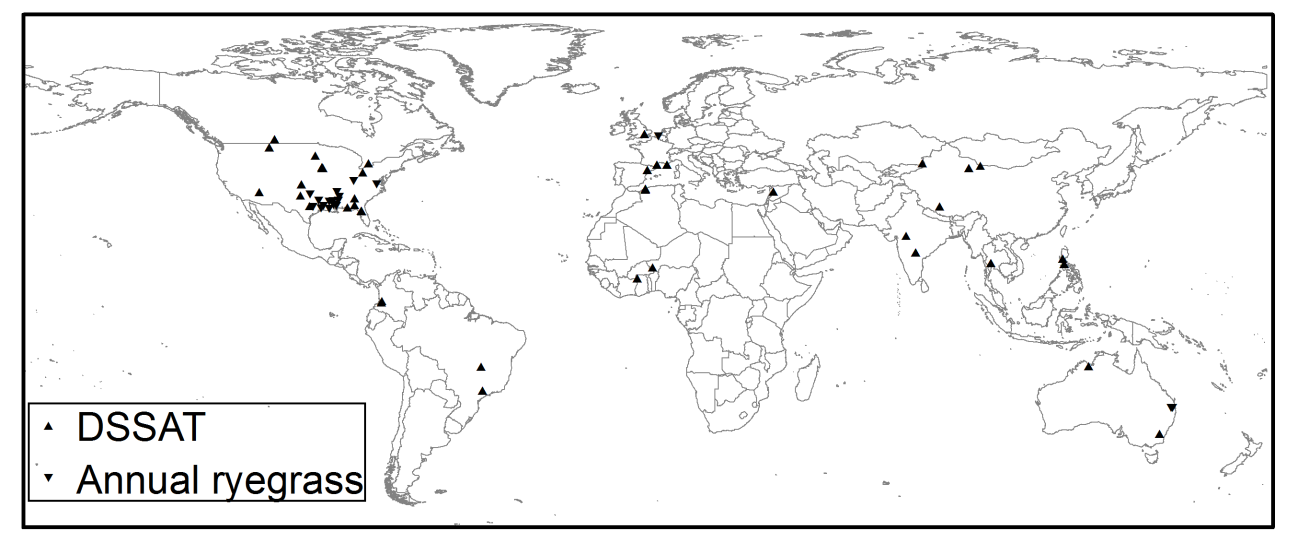


**Kim et al. Fig E1.**

**Kim et al. Fig E2.**
